# Tautomeric and Microscopic Protonation Equilibria of Anthranilic Acid and Its Derivatives

**DOI:** 10.1007/s10953-014-0190-3

**Published:** 2014-06-18

**Authors:** Lidia Zapała, Elżbieta Woźnicka, Jan Kalembkiewicz

**Affiliations:** Department of Inorganic and Analytical Chemistry, Faculty of Chemistry, Rzeszow University of Technology, 6 Powstańców Warszawy Ave., 35-959 Rzeszow, Poland

**Keywords:** Anthranilic acid, *N*-methylanthranilic acid, *N*-phenylanthranilic acid, Microconstants, Tautomerization constant, Dissociation constant

## Abstract

The acid–base chemistry of three zwitterionic compounds, namely anthranilic (2-aminobenzoic acid), *N*-methylanthranilic and *N*-phenylanthranilic acid has been characterized in terms of the macroconstants *K*_a1_, *K*_a2_, the isoelectric point p*H*_I_, the tautomerization constant *K*_z_ and microconstants *k*_11_, *k*_12_, *k*_21_, *k*_22_. The potentiometric titration method was used to determine the macrodissociation constants. Due to the very poor water solubility of *N*-phenylanthranilic acid the dissociation constants p*K*_a1_ and p*K*_a2_ were determined in MDM–water mixtures [MDM is a co-solvent mixture, consisting of equal volumes of methanol (MeOH), dioxane and acetonitrile (MeCN)]. The Yasuda–Shedlovsky extrapolation procedure has been used to obtain the values of p*K*_a1_ and p*K*_a2_ in aqueous solutions. The p*K*_a1_ and p*K*_a2_ values obtained by this method are 2.86 ± 0.01 and 4.69 ± 0.03, respectively. The tautomerization constant *K*_z_ describing the equilibrium between unionized form ⇌ zwitterionic form was evaluated by the *K*_z_ method based on UV–VIS spectrometry. The method uses spectral differences between the zwitterionic form (found at isoelectric pH in aqueous solution) and the unionized form (formed in an organic solvent of low dielectric constant). The highest value of the *K*_z_ constant has been observed in the case of *N*-methylantranilic acid (log_10_ *K*_z_ = 1.31 ± 0.04). The values of log_10_ *K*_z_ for anthranilic and *N*-phenylanthranilic acids are similar and have values of 0.93 ± 0.03 and 0.90 ± 0.05, respectively. The results indicate that the tested compounds, in aqueous solution around the isoelectric point pH_I_, occur mainly in the zwitterionic form. Moreover, the influence of the type of substituent and pH of the aqueous phase on the equilibrium were analyzed with regard to the formation and the coexistence of different forms of the acids in the examined systems.

## Introduction

Ampholytes are molecules containing both one (or more) acidic and one (or more) basic groups. They are encountered in drug research either as drugs or their metabolites. Ampholytes account for almost 15 % of ionizable drugs listed in the World Drug Index [1]. They can be classified into two groups, namely ordinary ampholytes and zwitterionic ampholytes. Ordinary ampholytes occur in a neutral and singly charged form. A study of their acid–base properties is usually not difficult and is carried out by the potentiometry or spectrophotometry. In order to describe the ionization process of ordinary ampholytes two (or more) p*K*_a_ values are sufficient and those values can be unambiguously assigned to the appropriate ionizable groups. Zwitterionic ampholytes are characterized by the fact that their two p*K*_a_ values are close to each other. Since it is not known which of the two dissociable groups dissociates first, the assignment of the p*K*_a_ values to the corresponding groups is difficult and sometimes even impossible [2].

Depending on the pH values of the media, zwitterionic compounds may exist in four different microforms, namely a cation (H_2_R^+^), zwitterion (HR^±^), neutral species (HR^0^) and anion (R^−^). Equilibria between these forms are expressed by the microconstants (p*k*_11_−p*k*_22_) and tautomeric ratio *K*_z_. Knowledge of microscopic constants and tautomeric ratios is thus required to provide information about specific sites of ionization.

The microscopic constants and tautomeric ratios describing zwitterionic ampholytes play an important role in understanding the ionic composition of many biologically active molecules and their physicochemical properties. The determination of these microconstants needs the combination of at least two experimental approaches. Besides, the potentiometric titration, which is used to obtain the macroconstants, the second method is typically spectroscopic (UV, NMR, CD, etc.) [3, 4].

Many methods can be used to investigate the microdissociation equilibria, however one can distinguish two main categories, namely the deductive and the direct methods [2, 5].

The deductive method is based on the assumption that blocking out an ionizable group in zwitterionic compounds by the chemical transformation unveils the micro-p*K*_a_ value of the other group without affecting it. The p*K*_a_ value of a derivative can be regarded as the relevant microconstant of the parent molecule.

Both the microconstant and the tautomeric constant can be determined also by using the direct method. This method is based on the determination of the *K*_z_ value by UV spectrometry, and is known as the *K*_z_ method. It is particularly suitable for the compounds in which protonation of both functional groups has an influence on spectral changes. This method was developed by Metzler and Snell and was used to determine the microscopic constants of pyridoxine in the dioxan–water system [6]. This approach was later applied to study of niflumic acid in methanol–water and dioxane–water systems [7]. For the past few years a new modification of the *K*_z_ method, which is based on multiwavelength spectrophotometric titration of the solution in various water–methanol systems, was also proposed [1, 8, 9]. The results obtained for niflumic acid and pyridoxine by the *K*_z_ methods before and after modification are in good agreement.

It should be noted that both the deductive and the direct methods are not precise and assumption-free simultaneously, what suggests that the results can only be regarded as estimated [2].

In this work our interest is focused on three zwitterionic compounds, namely anthranilic (2-aminobenzoic acid), *N*-methylanthranilic and *N*-phenylanthranilic acids. These compounds play very important roles in biological and chemical processes. Anthranilic acid is the precursor of l-tryptophan in plants, yeast and bacteria [10–12] and is also involved in the biosynthesis of phytohormones (auxins) and their precursors [13, 14]. It stimulates milk secretion in female mammals (it is called vitamin L1). Furthermore, anthranilic acid is widely used in the production of synthetic dyes [15]. Both anthranilic acid and *N*-phenylanthranilic acid are the starting materials for the synthesis of biologically active compounds and medicaments [16–19]. Their complexing properties are also used for metal ion determination [20]. In addition, it was shown that *N*-methylation of anthranilic acid to *N*-methylanthranilic acid is the first pathway-specific reaction in acridone alkaloid biosynthesis [21, 22]. Because of the widespread use of these compounds, the complete physicochemical characterization, which is based on the dissociation equilibrium, seems to be necessary. Hence, we have determined values of the macroconstants *K*_a1_ and *K*_a2_, the isoelectric point pH_I_, the tautomerization constant *K*_z_ and microconstants *k*_11_, *k*_12_, *k*_21_ and *k*_22_ by employing potentiometry and spectrophotometry. Moreover, we have analyzed the influence of the type of substituent and pH of the aqueous phase on the equilibrium with regard to the formation and coexistence of different forms of acids in the examined systems.

## Experimental

### Materials

All chemicals were used without further purification. Anthranilic (purity ≥98 %) and *N*-phenylanthranilic (purity ≥98 %) acids were purchased from Sigma–Aldrich. *N*-methylantranilic (purity 99 %) was obtained from Fluka. The HCl (1 mol·dm^−3^ ±0.045 %) and KOH (1 mol·dm^−3^ ±0.13 %) stock solutions were purchased from POCh Gliwice (Poland) as a TitraQuick^(^™^)^ Plus NIST concentrate for the preparation of standard solutions. The appropriate KOH solutions were obtained by dilution of the concentrate and then were standardized with a standard potassium hydrogen phthalate. The concentrations of HCl solutions were determined by potentiometric titrations using Gran’s method. Potassium hydrogen phthalate (purity ≥99.5 %) and KCl (purity ≥99.5 %) were from POCh Gliwice (Poland). Potassium hydrogen phthalate was dried at 110 °C before use. Methanol (HPLC grade, purity ≥99.9 %), acetonitrile (HPLC grade, purity ≥99.8 %), and dioxane (HPLC grade, purity ≥99.8 %) were purchased from Merck.

### Potentiometric Apparatus

The free hydrogen-ion concentration was measured via electromotive force by means of a CPI-505 pH-meter (Elmetron) (*E* ± 0.2 mV) with OSH 10-00 combined electrode (Metron). All potentiometric titrations (in aqueous solutions and in MDM–water mixtures) were performed in a thermostated double-walled vessel of 100 cm^3^ at 25.0 ± 0.1 °C under nitrogen atmosphere. The constant temperature in the vessel during the titration was maintained externally with a thermostat (P5 E1 Funke Medingen) equipped with a water bath. A digital automatic burette (VITLAB) was calibrated by weighing the doubly distilled and purified water with a precision of ±0.02 % in added volume over the whole volume range.

### Potentiometric p*K*_a_ Determination in Aqueous Solution

The dissociation constants were determined on the basis of the data obtained by potentiometric titration. 30.00 cm^3^ of anthranilic acid solution at a concentration of 0.010 mol·dm^−3^ was pre-acidified with HCl solutions to pH about 2.5 and then titrated with 0.0982 mol·dm^−3^ KOH solution to a pH of about 11.0. The titrations were carried out in 0.15 mol·dm^−3^ KCl solution (to adjust ionic strength, which not only mimicks the ionic strength of blood but greatly simplified the correction of the pH for its ionic strength dependence). The pH-metric titration procedure has previously been described in detail [23]. Titration of *N*-methylanthranilic acid solution was performed in analogy with the anthranilic acid solution but the *N*-methylanthranilic acid concentration was 0.002 mol·dm^−3^ and the concentration of the titrant (KOH solution) was 0.0196 mol·dm^−3^.

For each sample three parallel measurements were performed and p*K*_a_ values were calculated using the procedure described in Sect. 2.5. Titration data and the appropriate statistical parameters on anthranilic acid were presented in [23] while these parameters for *N*-methylanthranilic acid are summarized in Table 1.

**Table 1:** Titration data used for calculation of the dissociation constants of *N*-phenylanthranilic acid and the statistical parameters: *r* ^2^ coefficient of determination; *R*-*factor* Hamilton R-factor; *S* residual sum of squares; *SE*(*y*) standard error of the estimate (root-mean-square deviation-RMSD)

Initial concentration of *N*-phenylanthranilic acid, *c* [mol·dm^−3^]	Investigated pH range	Number of experimental point	Reliability of parameters estimation by a statistical analysis
0.002	2.69–8.94	86	*r* ^2^ = 0.99970
			*R*-*factor* = 1.10 %
			*S* = 0.1914
			*SE* (*y*) = 0.04861
0.002	2.68–8.79	85	*r* ^2^ = 0.99965
			*R*-*factor* = 1.17 %
			*S* = 0.2200
			*SE* (*y*) = 0.05180
0.002	2.69–8.77	85	*r* ^2^ = 0.99981
			*R*-*factor* = 0.81 %
			*S* = 0.1198
			*SE* (*y*) = 0.03823

### Potentiometric p*K*_a_ Determination in MDM–Water Mixtures

For the calibration of the glass electrode in MDM–water mixtures (MDM is a co-solvent, consisting of equal volumes of methanol (MeOH), dioxane and acetonitrile (MeCN)), HCl solutions of known concentrations containing 30.0–70.0 % (v/v) MDM were titrated with a 0.0107 mol·dm^−3^ KOH solution. Potassium hydroxide and hydrochloric acid solutions were prepared in each MDM–water system by suitable dilution of 1.000 mol·dm^−3^ standardized solution [24]. Titrations were conducted in the pH range 2.0–12.0 at temperature 25.0 ± 0.1 °C at the constant ionic strength (0.15 mol·dm^−3^ KCl). The Nernst equation parameters of the glass electrode for previously mentioned solvent mixtures were evaluated using a set of experimental data (emf, titrant volume). Calibration of the electrode in each system was repeated at least twice. The changes in p_s_ *K*_w_ values with mass fraction of MDM were evaluated on the basis of the polynomial equation shown in [24]. The values are presented in Table 2.

**Table 2:** Values of the parameters characterizing the MDM–water mixtures (*A* and *B* are the Debye–Hückel constants) and p_s_ *K*_a1_ and p_s_ *K*_a2_ values for *N*-phenylanthranilic acid in MDM–water systems

% (wt) MDM	*A*	*a*_0_ *B*	p_s_ *K*_w_	p_s_ *K*_a1_	p_s_ *K*_a2_	Coefficient of determination *r* ^2^	Number of experimental points, *N*
27.01	0.669	1.620	14.399	2.82 ± 0.02	5.05 ± 0.01	0.9998	77
36.53	0.760	1.683	14.675	2.77 ± 0.03	5.17 ± 0.01	0.9996	93
46.33	0.874	1.755	14.939	2.71 ± 0.02	5.42 ± 0.01	0.9997	88
56.43	1.024	1.841	15.129	2.64 ± 0.02	5.66 ± 0.01	0.9995	98
66.83	1.224	1.944	15.168	2.57 ± 0.01	5.96 ± 0.01	0.9998	96

The potentiometric titrations of *N*-phenylanthranilic acid were conducted under the same conditions as the electrode calibration. Six separate semiaqueous solutions were prepared containing 30.0–70.0 % (v/v) of MDM, 0.001 mol·dm^−3^ *N*-phenylanthranilic acid, and 0.15 mol·dm^−3^ KCl. The solutions were preliminary acidified by HCl solution and titrated with 0.0107 mol·dm^−3^ KOH solution (prepared in each MDM–water system by suitable dilution of a 1.000 mol·dm^−3^ standardized solution [24] ) to pH about 11. Titrations of each system were repeated in triplicate.

The activity coefficients *γ*_*i*_ in MDM–water mixture were calculated by using the Debye–Hückel equation Eq. 1:

1 $$ \log_{10}  \gamma_{i} = \frac{{Az^{2} \sqrt I }}{{1 + a_{0} B\sqrt I }} $$

where *I* is the ionic strength (*I* = 1/2 ∑ *c*_*i*_ *z*_*i*_^2^)and *c*_*i*_ is the molar concentration of ion *i* and *z*_*i*_ is its charge number; *A* and *B* are the Debye–Hückel constants, *a*_0_ the parameter dependent on the ion size, which was assigned a value fixed by the Bates–Guggenheim convention [25] extended to solvents of relatively moderate permittivity.

The values of the constant *A* in the Debye–Hückel equation for each investigated solution have been calculated separately using Eq. 2 [26]. The dielectric constants *ε* and density *ρ* of MDM–water mixtures were interpolated based on the data presented in [24].

2 $$ A = \frac{{F^{3} }}{{4\pi N_{A} \ln 10}}\left( {\frac{\rho }{{2\varepsilon^{3} R^{3} T^{3} }}} \right)^{1/2} $$

where *F* is the Faraday constant, *N*_A_ the Avogadro constant, *R* the gas constant, *T* the absolute temperature, *ε* and *ρ* are the dielectric constant and the density of MDM–water mixtures, respectively.

Values of *a*_0_ *B* were calculated based on Eq. 3 [27]:

3 $$ a_{0} B = 1.5\sqrt {\frac{{\varepsilon_{w} \rho }}{{\varepsilon \rho_{w} }}} $$

where *ε*_w_ and *ρ*_w_ denote the dielectric constant and the density of water, respectively.

### Analysis of Titration Curves

The p*K*_a_ (for anthranilic and *N*-methylanthranilic acid) and p_s_ *K*_a_ (for *N*-phenylanthrnilic acid) can be deduced from the half-neutralization point of the titration curve but it is advantageous to determine the ionization constant from a difference curve (called formation curve or Bjerrum plot). It is the plot of $\bar{n}_{\rm {H}}$ (the average number of bound protons) against pH. For diprotic substances $\bar{n}_{\rm {H}}$ is defined by Eq. 4 [26]:

4 $$ \bar{n}_{\text{H}} = 2 + \frac{{ A -  c_{\text{b}} V - \left( {10^{{ - {\text{pH}}}} - 10^{{ - \left( {{\text{p}}K_{w} + {\text{pH}}} \right)}} } \right) (V_{0} + V)}}{L} $$

where *A* is the amount of added HCl [mmol], *c*_b_ the concentration of the titrant (KOH), *V* is the volume of the titrant (potassium hydroxide) added during the titration [mL], *V*_0_ is the initial volume of the solution [mL] and is *L* the total amount of titrated amino acid [mmol].

Theoretically, the fraction of the bounded protons for a diprotic acid can be evaluated based on Eq. 5 [28]:

5 $$ \bar{n}_{\text{H}} = \frac{{10^{{ - ({\text{pH}} - {\text{p}}K_{{{\text{a}}1}} )}} + 2 \times 10^{{ - 2{\text{pH}}}} }}{{10^{{ - 2{\text{pH}}}} + 10^{{ - \left( {{\text{pH}} + {\text{p}}K_{{{\text{a}}1}} } \right)}} + 10^{{ - \left( {{\text{p}}K_{{{\text{a}}1}} + {\text{p}}K_{{{\text{a}}2}} } \right)}} }} $$

Subsequently, the experimental titration data were converted into $\bar{n}_{\rm {H}}$ using Eq. 4 and fitted to the theoretical function defined by Eq. 5 using minimalization of the sum of residuals squares *S* between the experimental $\bar{n}_{\text{H,exp}}$ and calculated $\bar{n}_{\text{H,cal}}$ values expressed by Eq. 6.

6 $$ S = \mathop \sum \nolimits (\bar{n}_{\text{H, exp}} -  \bar{n}_{\text{H, cal}} )^{2} $$

A weighted non-linear least-squares regression method was used to provide a fast and reliable refining of p*K*_a1_ and p*K*_a2_ values. All calculations were performed using Microsoft Excel. Such data processing, based on potentiometric titrations, is recommended by many authors [28–31] who also give the exact description of how to implement all of these equations in a spreadsheet.

### Yasuda–Shedlovsky Procedure

*N*-phenylanthranilic acid is poorly soluble in water and its solubility in the methanol–water system is also limited; therefore, the dissociation constants p*K*_a1_ and p*K*_a2_ were determined in the MDM–water system. The apparent dissociation constant values (p_s_ *K*_a1_ and p_s_ *K*_a2_) were determined potentiometrically as described above and aqueous p*K*_a1_ and p*K*_a2_ values were obtained by extrapolation of the p_s_ *K*_a1_ and p_s_ *K*_a2_ values to 0 % of MDM with the Yasuda–Shedlovsky procedure expressed by Eq. 7:

7 $$ {\text{p}}_{\text{s}} K_{\text{a}} + { \log }_{ 10} \left[ {{\text{H}}_{ 2} {\text{O}}} \right] \, = a/\varepsilon + b $$

where [H_2_O] is the molar water concentration in the given solvent mixture and *ε* is the dielectric constant of the co-solvent mixture.

### Determination of Microconstant Values

A Beckman DU-640 single beam spectrophotometer was used to record the electronic absorption spectra in the range of 200–800 nm, using 1 cm path length quartz cuvettes, at room temperature. UV spectroscopy was used to determine the tautomerization microconstant *K*_z_ values for the equilibrium HR^0^ (unionized form) *⇌* HR^±^ (zwitterionic form). The method is based on the spectral differences between the zwitterionic form of the molecule (found predominantly at the isoelectric pH in aqueous solution) and the unionized form (found predominantly in an organic solvent of low dielectric constant, such as methanol).

The stock solutions of anthranilic and *N*-methylanthranilic acid (0.040 mol·dm^−3^) were prepared in methanol and of *N*-phenylanthranilic (3.0 × 10^−3^ mol·dm^−3^) in MDM. 1 cm^3^ of each of these solutions was diluted to 200 cm^3^ with methanol or Britton–Robinson buffer (pH = 3.51, 3.94, 3.78 for anthranilic, *N*-methylantranilic, *N*-phenylanthranilic acids, respectively). Then, mixtures of the solutions of each acid in methanol and buffer, containing 0–100 % methanol (in steps of 10 %), were prepared. Next, the absorption spectra of these solutions were recorded and tautomerization microconstant were calculated from the spectroscopic data based on Eq. 8 [7]. Each experiment was repeated at least three times.

8 $$ K_{{{\text{z(\% )}}}}  { = }\frac{{A_{{{\text{HR}}^{0} }} { - }A_{\% } }}{{A_{\% } { - }A_{{{\text{HR}}^{ \pm } }} }} $$

where *K*_z(%)_ tautomerization constant in a given % solvent mixture, *A*_%_ the absorbance of the compound in the given % solvent mixture, $A_{{\rm {HR}^{0} }}$ the absorbance of the compound in the pure organic solvent and $A_{{\rm {HR}^{ \pm } }}$ the absorbance of the compound in aqueous buffer solution at the isoelectric pH.

## Results and Discussion

The anthranilic acid derivatives, which are zwitterionic compounds, were studied using infrared and ultraviolet absorption spectra. The results have provided evidence that these compounds may exist in the neutral or zwitterionic forms and that the tautomeric equilibrium HR^0^ *⇌* HR^±^ is strongly solvent dependent. The increase of the solvent dielectric constant values influences the stability of the HR^±^ form. Furthermore, these compounds, in the inert solvent, exist in the quasi-zwitterionic form with a strong intramolecular –COOH···NR_2_– hydrogen bond and their zwitterionic form is stable only in a highly polar solvent [32].

**Fig. 1:**
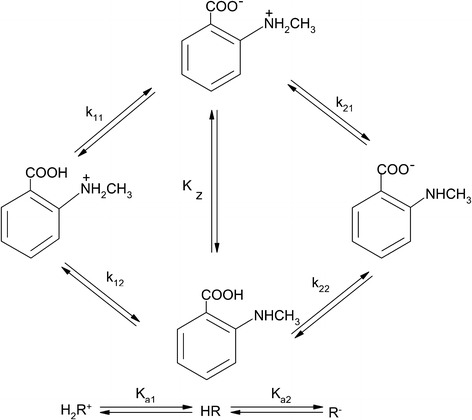
Ionization of *N*-methylanthranilic acid

The equilibria of the tested compounds (anthranilic, *N*-methylantranilic and *N*-phenylanthranilic acids) in the aqueous solution with the appropriate macro- and microdissociation constants can be presented as shown below in Fig. 1.

The macroscopic ionization constants for this two-step procedure are defined by:

9 $$ K_{\text{a1}}  { = }\frac{{[{\text{HR}}][{\text{H}}^{ + } ]}}{{[{\text{H}}_{2} {\text{R}}^{ + } ]}} $$

and

10 $$ K_{\text{a2}}  { = }\frac{{[{\text{R}}^{ - } ] [ {\text{H}}^{ + } ]}}{{ [ {\text{HR]}}}} $$

where [HR] = [HR^±^] + [HR^0^] is the the total concentration of zwitterionic and neutral species, which are indistinguishable by the acid–base titration.

The microconstants provide information on the individual proton binding sites and their interaction with other sites in the molecule.

The relationships between the micro- and macroconstants have been reported in the literature [5, 33]:

11 $$ K_{\text{a1}} = k_{ 1 1} + k_{ 1 2} $$

12 $$ \frac{1}{{K_{\text{a2}} }} { = }\frac{1}{{k_{ 2 1} }} { + } \frac{1}{{k_{ 2 2} }} $$

13 $$ K_{\text{a1}} K_{\text{a2}} = k_{ 1 1} k_{ 2 1}  = k_{ 1 2} k_{ 2 2} $$

14 $$ K_{z} = \frac{{k_{ 1 1} }}{{k_{ 1 2} }} = \frac{{k_{ 2 2} }}{{k_{ 2 1} }} $$

The determination of the macroconstants p*K*_a1_ and p*K*_a2_ for compounds that are soluble in water is not problematic; therefore, a simple potentiometric titration is sufficient to achieve this objective. In this way the values of dissociation constants of anthranilic and *N*-methylantranilic acids were determinated and are presented in Table 3. The analysis of titration data was performed using Microsoft’s Excel computer package. The values were then refined by a weighted nonlinear last-squares procedure using the Solver program from Microsoft Excel.

**Table 3:** Dissociation macroconstants (*K*_a1_ and *K*_a2_), microconstants (*k*_11_, *k*_12_, *k*_21_ and *k*_22_), tautomeric constant (*K*_z_) and isoelectric point (p*H*_I_) of anthranilic, *N*-methylanthranilic and *N*-phenylanthranilic acids

Constants	Anthranilic acid [23]	*N*-methylanthranilic acid	*N*-phenylanthranilic acid
p*K*_a1_	2.18 ± 0.05	2.52 ± 0.02	2.86 ± 0.01
p*K*_a2_	4.84 ± 0.01	5.35 ± 0.01	4.69 ± 0.03
log_10_ *K*_z_	0.93^a^ ± 0.03	1.31^a^ ± 0.04	0.90^a^ ± 0.05
p*k*_11_	2.25 ± 0.05	2.54 ± 0.02	2.91 ± 0.01
p*k*_12_	3.18 ± 0.05	3.85 ± 0.02	3.80 ± 0.01
p*k*_21_	4.79 ± 0.01	5.33 ± 0.01	4.64 ± 0.03
p*k*_22_	3.86 ± 0.01	4.02 ± 0.01	3.74 ± 0.03
p*H*_I_	3.51 ± 0.02	3.94 ± 0.02	3.78 ± 0.02

^a^Values obtained by the extrapolation based on the Eq. 15 (refer to the pure water as a solvent)

For compounds which are practically insoluble in water (e.g. *N*-phenylanthranilic acid), the determination of the macroconstants p*K*_a1_ and p*K*_a2_ is much more difficult. In such cases potentiometric titration in the methanol–water system is usually applied. The results obtained on the basis of the titration of the series of solutions in the methanol–water system were presented earlier [34]. However, the solubility of this compound in systems containing less than 50 % of methanol is incomplete and so the results may be subject to some errors; hence, we decided to repeat the study of this compound in MDM–water systems. MDM is a co-solvent mixture, consisting of equal volumes of methanol (MeOH), dioxane and acetonitrile (MeCN). This mixture improves the solubility of the hydrophobic compounds and is still a good solvent for polar molecules and fulfils all the requirements that are needed for its application in the pH-metric method. The validation of the p*K*_a_ determination in MDM–water mixtures was presented in [24].

Subsequently, to determine the best aqueous p*K*_a1_ and p*K*_a2_ values, the refined values of p_s_ *K*_a_ were extrapolated to zero co-solvent by the Yasuda–Shedlovsky procedure expressed by Eq. 7. The values of p_s_ *K*_a_ for *N*-phenylanthranilic acid with the statistical parameters are presented in Table 2.

The potentiometric studies show that, with the increase of the content of the organic solvent, the p_s_ *K*_a2_ (the apparent dissociation constant) values also increase. Similar observations were also made for other anthranilic acid derivatives (unpublished studies). Addition of the organic solvent causes the suppression of dissociation, and usually indicates the presence of an acidic center, while the decrease of p_s_ *K*_a1_ values with an increase of the content of the organic solvent may indicate the presence of a basic center [35].

**Fig. 2:**
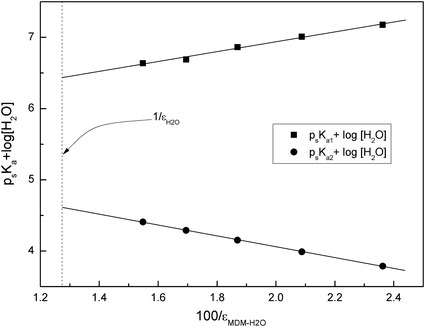
Yasuda–Shedlovsky plot for *N*-phenylanthranilic acid in MDM–water mixtures for determination of the p*K*_a1_ and p*K*_a2_ values

The Yasuda–Shedlovsky plot for *N*-phenylanthranilic acid in the MDM–water system is shown in Fig. 2.

It should be noted that the slope of the Yasuda–Shedlovsky extrapolation for the basic group is negative while the slope corresponding to the acidic group is positive. This trend is observed in most of the ampholytes [9, 27, 29]. The p*K*_a1_ and p*K*_a2_ values obtained by this method are 2.86 ± 0.01 and 4.69 ± 0.03, respectively. The isoelectric point pH_I_ was identified at pH = 3.78 ± 0.02. Hence, the p*K*_a_ values obtained in this way could be assigned to amino and carboxylic groups, respectively.

**Fig. 3:**
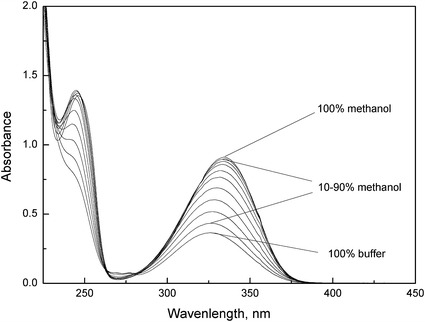
Absorption spectra of anthranilic acid (*c* = 2.0 × 10^−4^ mol·dm^−3^) in the different methanol–buffer mixtures (0–100 % (v/v))

**Fig. 4:**
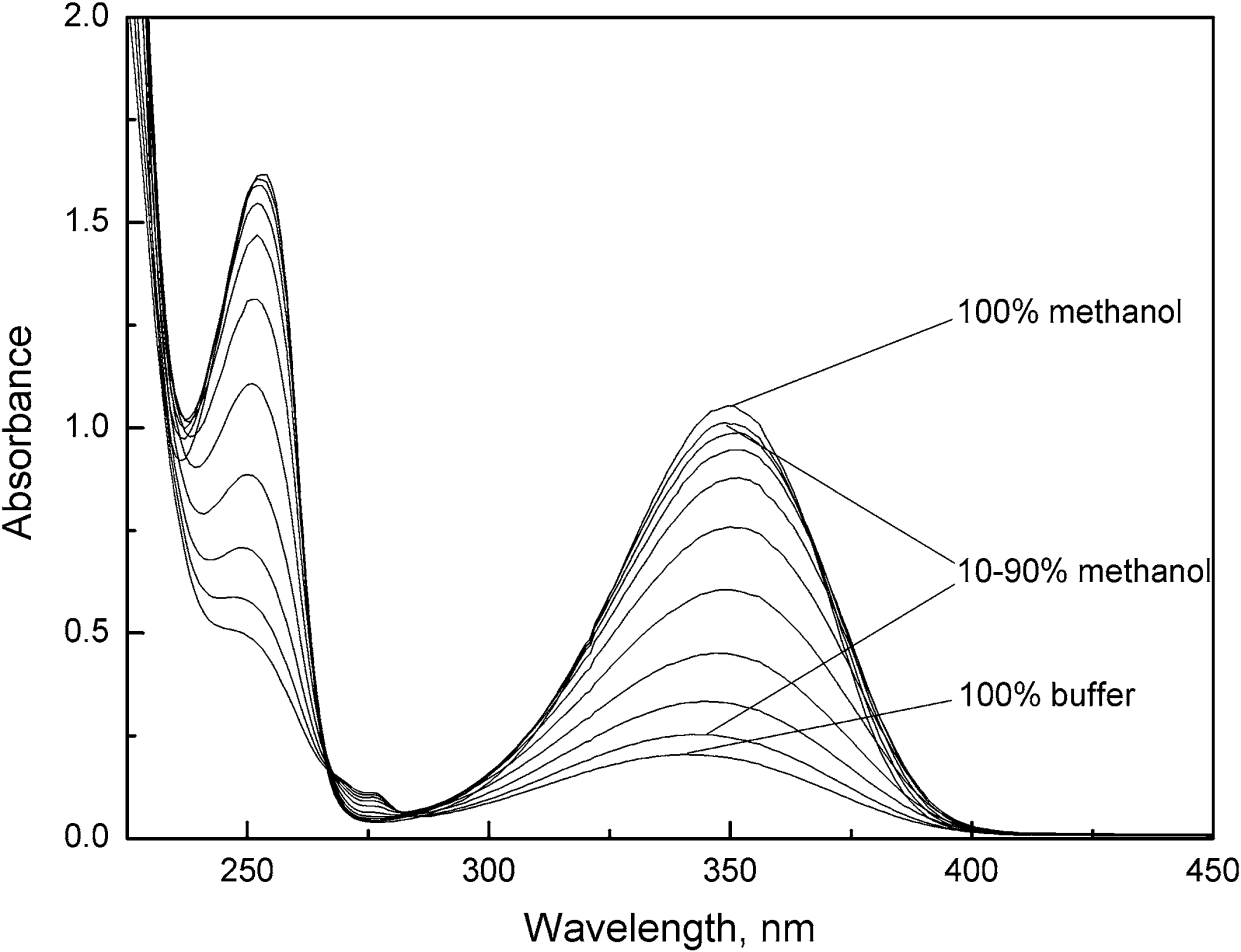
Absorption spectra of *N*-methylanthranilic acid (*c* = 2.0 × 10^−4^ mol·dm^−3^) in the different methanol–buffer mixtures (0–100 % (v/v))

**Fig. 5:**
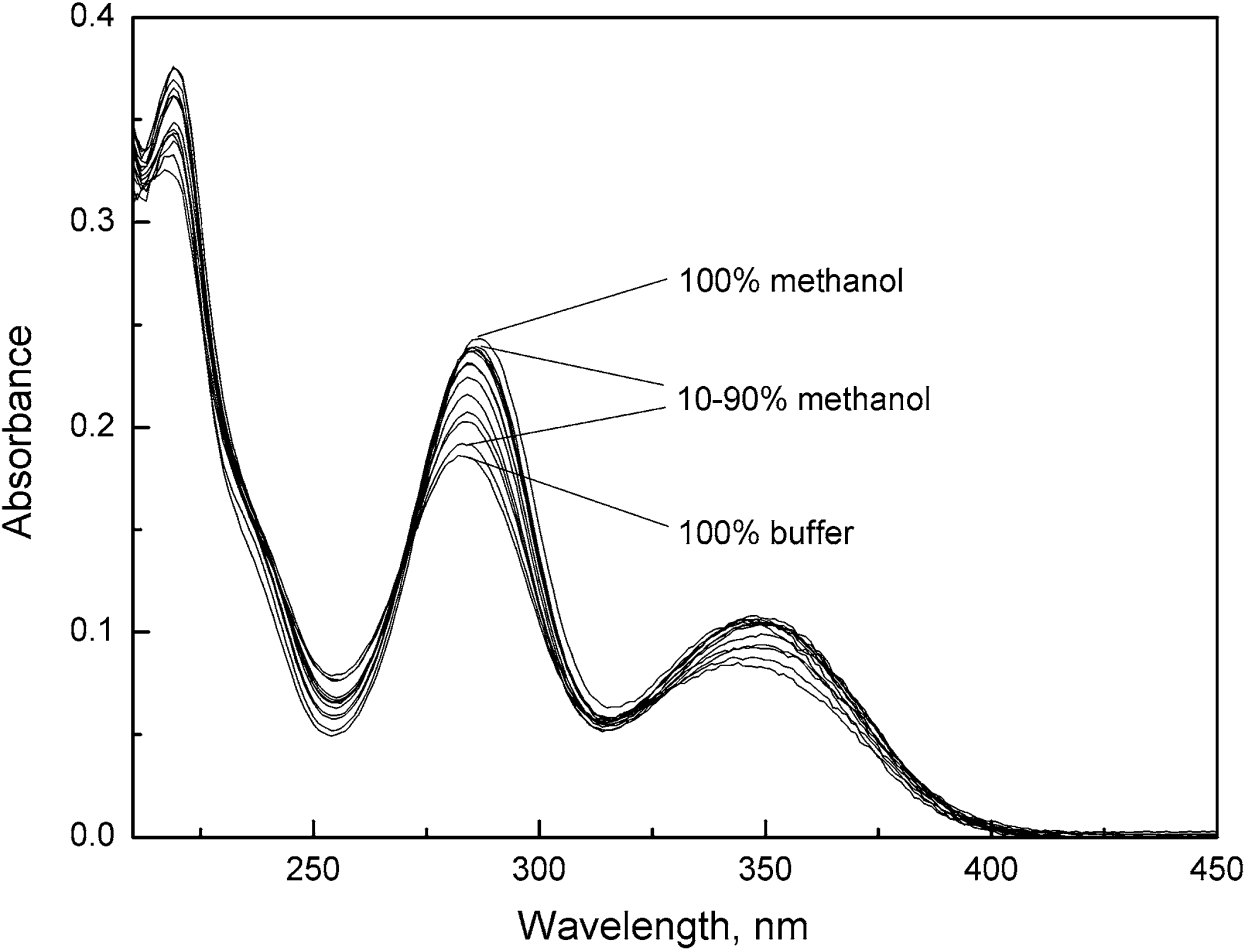
Absorption spectra of *N*-phenylanthranilic acid (*c* = 1.5 × 10^−5^ mol·dm^−3^) in the different methanol–buffer mixtures (0–100 % (v/v))

Anthranilic, *N*-methylantranilic, and *N*-phenylanthranilic acids belong to the group of zwitterionic compounds with two ionizable groups connected to the chromophores [5]. For that reason selective monitoring of any ionized group is impossible as is application of the deductive method. The absorption spectra of the studied compounds in the solutions containing different amounts of methanol and water (buffer at the pH of the isoelectric point) were performed to determine the values of tautomeric ratio *K*_z_ and microconstants *k*_11_−*k*_22_ [7, 9, 36], and are presented in Figs. 3, 4, 5.

The absorption spectra of all studied compounds are characterized by two bands. A longer wavelength band for anthranilic acid has a maximum at 326 nm (in Britton–Robinson buffer) and in the case of *N*-methylanthranilic and *N*-phenylanthranilic acids, this is red shifted to about 341 and 346 nm. In turn, a shorter wavelength band for anthranilic and *N*-methylanthranilic acids have a maximum at about 240 and 247 nm, respectively that is not very distinct. On the other hand, in the case of *N*-phenylanthranilic acid, this band is very distinct and the maximum is red shifted to about 283 nm. As described in the literature [37, 38], the shorter wavelength band is ascribed to the *π*–π* transition of the benzenoid system, whereas the longer wavelength band can be attributed to the *π*–π* transition within the heterocyclic moiety (IHB ring) of the studied compounds. It was also observed that, with an increase of methanol content, the maximum in the shorter wavelength band is more distinct both for anthranilic and *N*-methylanthranilic acids. Furthermore, the addition of methanol causes all absorption spectra to be red shifted relative to those in Britton–Robinson buffers.

Moreover, all of the absorption spectra show an isosbestic point, which indicates that only two species are in equilibrium under the experimental conditions. The presence of the sharp isosbestic point allows the precise determination of the *K*_z_ constant value.

Assuming an exponential relationship between the *K*_z%_ values and the percentage of methanol, the aqueous *K*_z_ value can be obtained from the intercept of the following equation [7]:

15 $$ { \log }_{ 10} K_{{{\text{z}}\% }} = WP + S $$

where *P* stands for wt% methanol; *W*, *S* are the slope and intercept, respectively.

When the *K*_z_ value is obtained by the fitting procedure, all microconstants can be calculated from *K*_z_, p*K*a_1_ and p*K*_a2_ based on Eqs. 11–14. It should be stressed that the ratio of HR^±^ to HR^0^ is independent of pH and is a characteristic feature of the individual amino acid. The obtained tautomerization constant *K*_z_ and microconstants (*k*_11_, *k*_12_, *k*_21_ and *k*_22_) values are summarized in Table 3.

The p*K*_a1_ and p*K*_a2_ values of anthranilic, *N*-methylantranilic and *N*-phenylanthranilic acids represent the acidity of the protonated amino and carboxyl groups, respectively. As presented in Table 3, significant differences are observed in the case of p*K*_a1_ as well as p*K*_a2_ values.

The acidity of the protonated amino group decrease in the series: anthranilic acid > *N*-methylanthranilic > *N*-phenylanthranilic.

The value of p*K*_a1_ for *N*-phenylanthranilic is relatively high in comparison with other examined acids. This is probably due to steric hindrance which impedes the dissociation of the protonated amino group.

The acidities of the carboxylic groups of the acids decrease in the order: *N*-phenylanthranilic > anthranilic acid > *N*-methylanthranilic.

The p*K*_a2_ value for *N*-methylanthranilic acid indicates that acidity of the carboxylic group decreases compared to anthranilic acid. The electron donating effect from methyl substitution to the amino group stabilizes the protonation state of the carboxyl group. On the other hand, substitution of the phenyl group (electron acceptor) to the amino group facilitates the dissociation of the carboxyl group, which in turn causes an increase in acidity.

The values of *K*_z_ for are very small in comparison with aliphatic amino acids.

On the other hand, the values of log_10_ *K*_z_ for 3-aminobenzoic and 4-aminobenzoic acids are equal 0.43 [5] and −0.93 [39], respectively. The obtained values of log_10_ *K*_z_ constants for the studied compounds are significantly higher. This is due to the preferred position of the amino and carboxyl groups in relation to each other and their strong interaction.

The highest value of *K*_z_ is observed in the case of *N*-methylantranilic acid (log_10_ *K*_z_ = 1.31). The value of this constant is higher than presented in the literature [32] (log_10_ *K*_z_ = 0.58). These different values of *K*_z_ probably result from the use of different methods for their determination (we used the direct method, and the authors of [32] the deductive method). The values of log_10_ *K*_z_ for anthranilic and *N*-phenylanthranilic acids are similar and slightly lower than the value of log_10_ *K*_z_ for *N*-methylantranilic acid. In turn, compared with niflumic acid (log_10_ *K*_z_ = 1.24) [7], the value of *K*_z_ for *N*-phenylanthranilic acid is lower. The higher value of the *K*_z_ constant for niflumic acid is connected with its structure resulting from the presence of the nitrogen atom in the benzene ring.

The microconstants relating to the same dissociation site (*k*_11_ and *k*_22_ or *k*_12_ and *k*_22_, respectively) have significantly different values. This means that the dissociation at one site causes a considerable decrease of the acidity of the other site (p*k*_11_ < p*k*_22_ and p*k*_12_ < p*k*_21_) [40]. The above observation can be a quantitative measure of the interaction between two sites when dissociation occurs at one of them. The values of this interactivity parameter ∆p*k* (∆p*k* = p*k*_22_−p*k*_11_ = p*k*_21_−p*k*_12_) are 1.61, 1.48 and 0.83 for anthranilic, *N*-methylantranilic and *N*-phenylanthranilic acids, respectively. The ∆p*k* values of anthranilic and *N*-methylantranilic acids are higher than that for *N*-phenylanthranilic acid. In turn, the ∆p*k* value of *N*-phenylanthranilic acid is comparable to that for niflumic acid (∆p*k* = 0.90) [7]. Despite the significant differences in the size of this parameter one can conclude that for all investigated compounds there are strong interactions between the two dissociation sites.

**Fig. 6:**
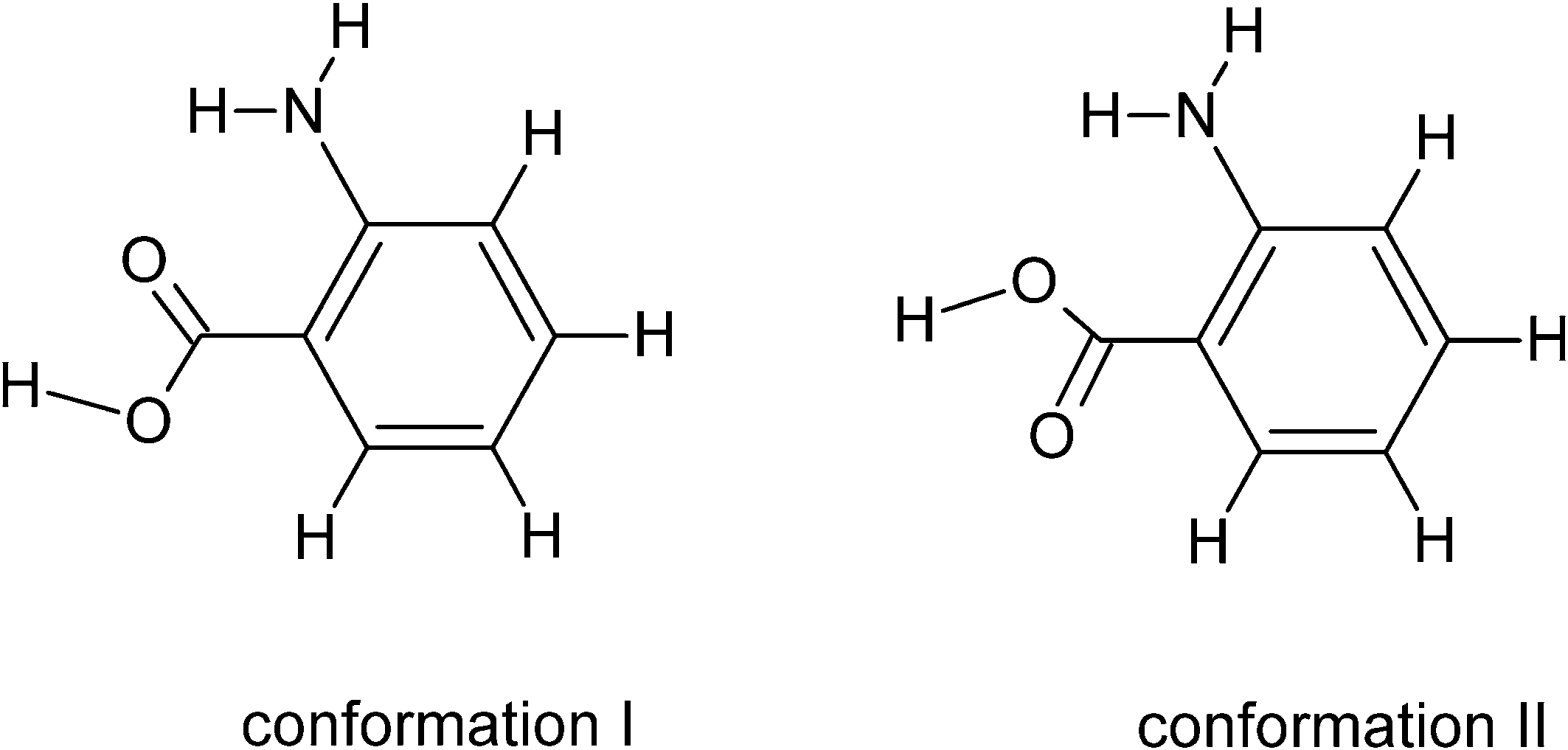
Geometric structure of anthranilic acid [41]

In comparison with the number of experimental studies on anthranilic acid and their derivatives, we can find very few theoretical studies on the effects of the solvents on the equilibria of aromatic amino acids, in particular, as regards the determination of tautomerization equilibria. In order to analyze the electronic structure of ortho-anthranilic acid, both in gas phase and aqueous solution, high-level ab initio methods were used. The calculations have shown that anthranilic acid can exists in the two lowest energy conformations called conformation I and conformation II (Fig. 6) [41].

The electronic properties of the two conformers are quite similar excluding the dipole moment. The dipole moment of conformation I is 1.29 Debye, whereas that of the conformation II is 2.46 Debye. Thus, the conformation I is found to be more stable, less polar but more polarizable than conformation II. Furthermore, conformation I is convenient for the formation of intramolecular N–H^**….**^O hydrogen bonds. Similar calculations were made for the rotameric conformations of three amino derivatives of anthranilic acid [42]. The results are in good agreement with those for anthranilic acid [41]. Besides, their study has shown higher stability for the rotameric conformation in which the carbonyl oxygen is near the nitrogen of the ortho-amino group. On the other hand, the creation of zwitterions is closely connected with the solvent effects [43]. It was concluded that the neutral forms of amino acid predominate in non-polar solvents while zwitterionic forms are found in polar solvents. The strong solvating power of water (in relation to another solvents like dimethyl sulphoxide and methanol) is capable to breaking the strong intramolecular charge transfer hydrogen bond between the COO^−^ and N^+^R_2_H groups. This is largely related with the ability of water to solvate both above groups while other solvents can only solvate one of them.

**Fig. 7:**
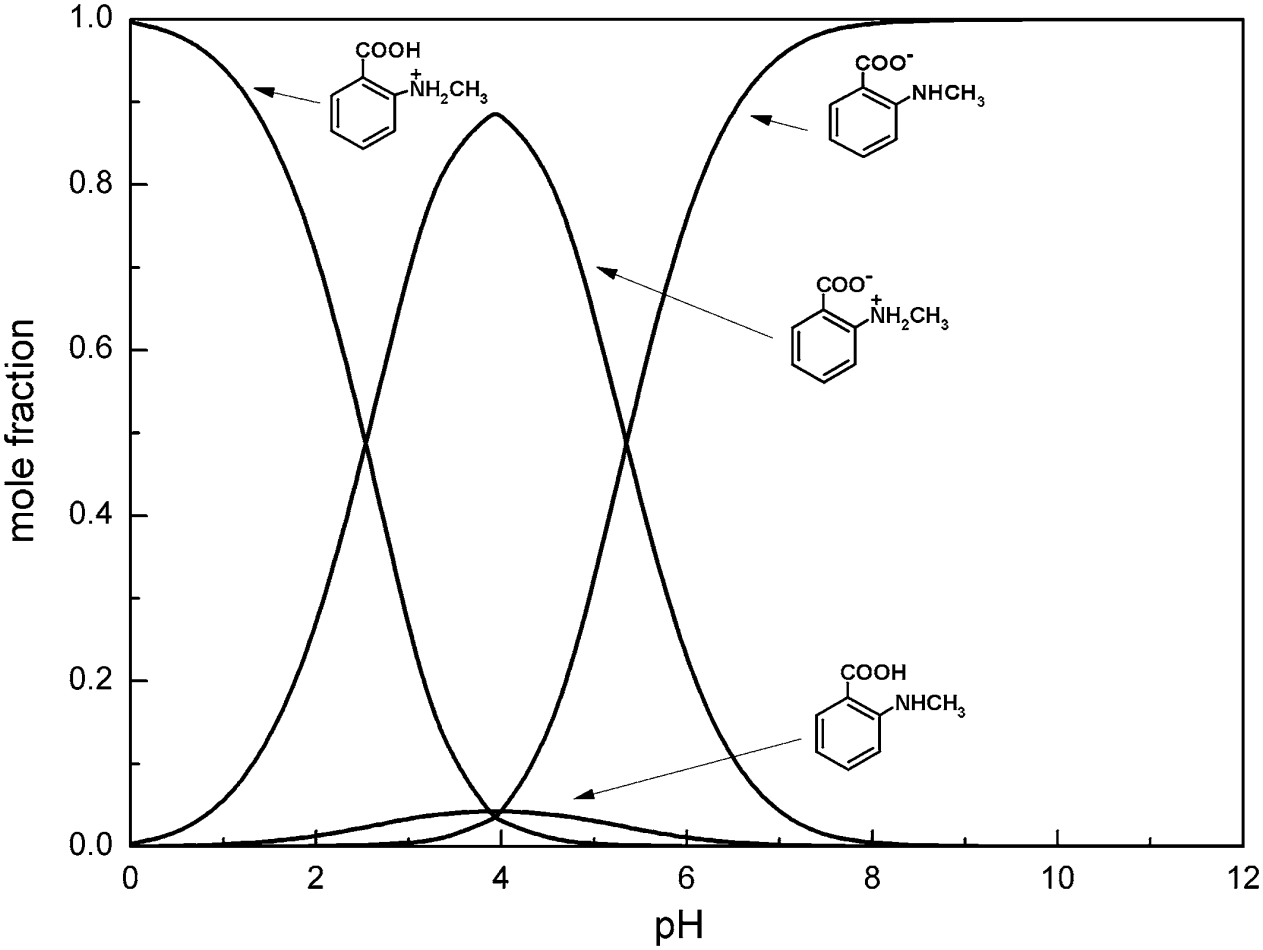
Microspeciation diagram of *N*-methylanthranilic acid in the aqueous solution

**Fig. 8:**
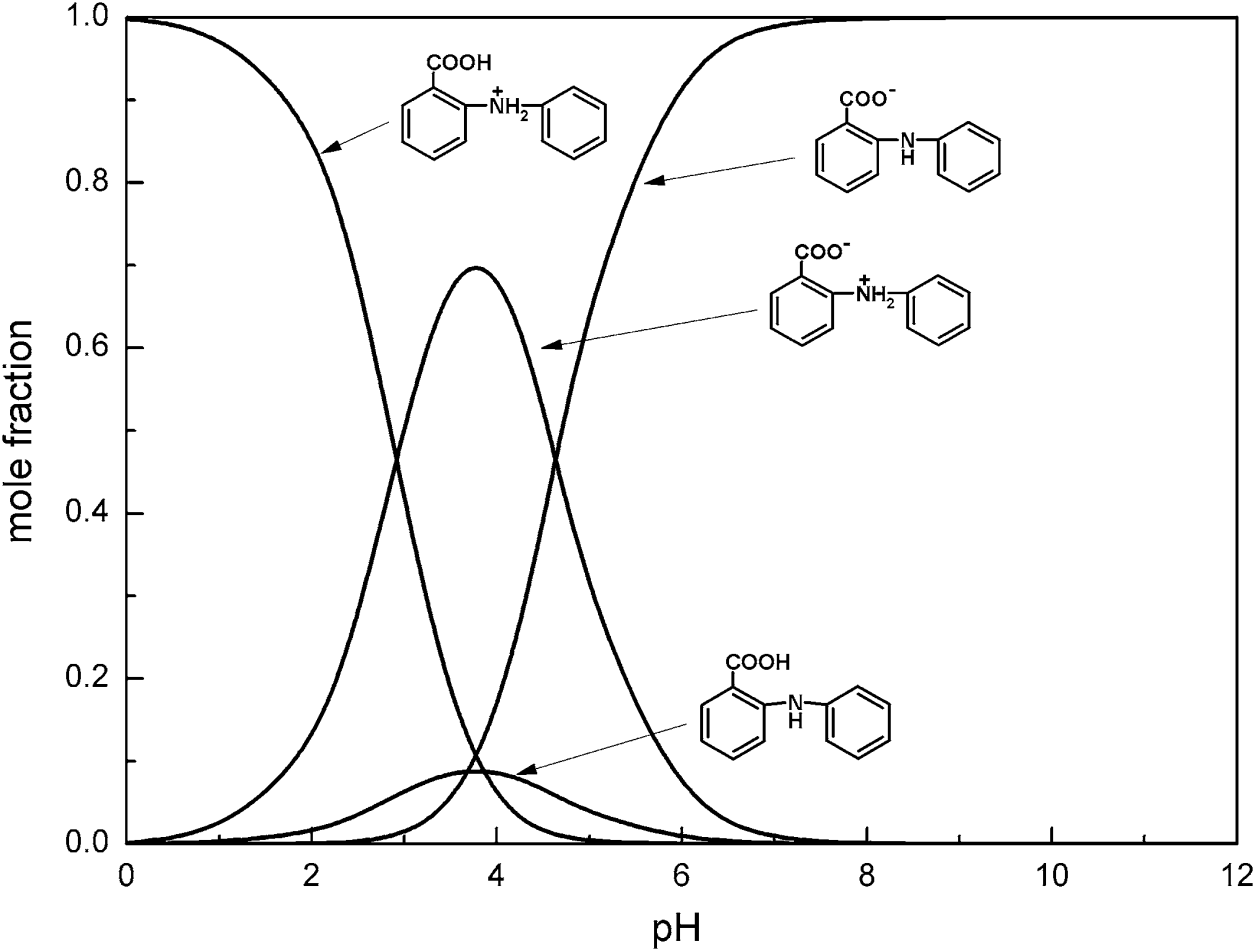
Microspeciation diagram of *N*-phenylanthranilic acid in the aqueous solution

The determination of the microconstants of studied compounds allows construction of the microspeciation diagrams, which are shown in Figs. 7, 8 and presented in the literature [23] for *N*-methylanthranilic, *N*-phenylanthranilic and anthranilic acids, respectively. The relative concentrations of microspecies in solutions can be calculated as described in the literature [23].

The diagrams show that the form H_2_R^+^ dominates at pH < 2.5. The zwitterionic microspecies HR^±^ predominates at pH_I_ for all studied compounds. The content of neutral form HR^0^ reaches a maximum also at the isoelectric point, but its amount is significantly lower than that of the zwitterionic form HR^±^. The highest content of the zwitterionic form (at isoelectric point) is observed for *N*-methylanthranilic acid (mole fraction 0.89) and the lowest for *N*-phenylanthranilic (mole fraction 0.71). In turn, the lowest content of the neutral form HR^0^ is observed for *N*-methylanthranilic acid (mole fraction 0.04) while the contents of this form of *N*-phenylanthranilic acid and anthranilic acid are comparable and equal to 0.09 and 0.10, respectively. When pH is higher than 5.5 the ionized form R^−^ is the dominant one for all tested compounds.

## Conclusion

The studied compounds, i.e. anthranilic acids, *N*-methylanthranilic and *N*-phenylanthranilic may exist either as zwitterions or uncharged molecules. The ratio of concentration of these two forms *K*_z_ is constant at a given temperature and may be evaluated by UV–VIS spectrometry (called the *K*_z_ method). This method uses spectral differences between the zwitterionic forms (found at isoelectric pH in aqueous solution) and the unionized form (formed in an organic solvent of low dielectric constant). The obtained values of *K*_z_ are very small in comparison with aliphatic amino acids. On the other hand, they are significantly higher than these obtained for the other amino benzoic acids (substituted in the meta- and para position) due to the preferred position of the amino and carboxyl groups in relation to each other and their strong interaction.

Our experiments show that the tested compounds in the aqueous solution around isoelectric pH occur mainly in the zwitterionic form.

The potentiometric titration method was used to determine the macrodissociation constant. The use of the MDM–water mixtures and Yasuda–Shedlovsky procedure allowed us to determine accurately the dissociation constants of *N*-phenylanthranilic acid.

The obtained results indicate that there are the significant differences in the values of macro- and microdissociation constants of the tested compounds and they result from their structural differences. Recapitulating, the potentiometry and spectrophotometric methods seem to be suitable for the study of intramolecular interactions of the aromatic amino acids containing both acidic and basic groups in the ortho position.
